# Post-traumatic peripheral vestibular disorders (excluding positional vertigo) in workers following head injury

**DOI:** 10.1038/s41598-021-02987-5

**Published:** 2021-12-06

**Authors:** Priyanka Misale, Fatemeh Hassannia, Sasan Dabiri, Tom Brandstaetter, John Rutka

**Affiliations:** Department of Otolaryngology-Head and Neck Surgery, Toronto General Hospital, University Health Network, University of Toronto, 200 Elizabeth Street, 8N-873, Toronto, ON M5G 2C4 Canada

**Keywords:** Diseases, Health occupations, Neurology, Signs and symptoms

## Abstract

Benign paroxysmal positional vertigo has typically been reported to be the most common cause of post-traumatic dizziness. There is however paucity in the literature about other peripheral vestibular disorders post-head injury. This article provides an overview of other causes of non-positional dizziness post-head trauma from our large institutional experience. The UHN WSIB Neurotology database (n = 4291) between 1998 and 2018 was retrospectively studied for those head-injured workers presenting with non-positional peripheral vestibular disorders. All subjects had a detailed neurotological history and examination and vestibular testing including video nystagmography, video head impulse testing (or a magnetic scleral search coil study), vestibular-evoked myogenic potentials, and audiometry. Imaging studies included routine brain and high-resolution temporal bone CT scans and/or brain MRI. Based on a database of 4291 head-injured workers with dizziness, 244 were diagnosed with non-positional peripheral vertigo. Recurrent vestibulopathy (RV) was the most common cause of non-positional post-traumatic vertigo. The incidence of Meniere’s disease in the post-traumatic setting did not appear greater than found in the general population. The clinical spectrum pertaining to recurrent vestibulopathy, Meniere’s disease, delayed endolymphatic hydrops, drop attacks, superior semicircular canal dehiscence syndrome, and uncompensated peripheral vestibular loss are discussed.

## Introduction

Organic causes for post-traumatic dizziness are primarily peripheral vestibular rather than central in origin^1^. Positional vertigo after head trauma is well documented; the most frequent peripheral form of vertigo being benign paroxysmal positional vertigo (BPPV) from a posterior semicircular response^2^. Acute vertigo from labyrinthine trauma usually resolves over weeks to months spontaneously or from central compensation (neuroplasticity)^3^. Failure to compensate for a traumatic vestibular loss remains another cause for prolonged dizziness in the head injured individual.

Recurrent attacks of spontaneous vertigo lasting minutes to hours following head trauma appear less frequent^1^. Considerations include a spectrum of clinical entities such as recurrent vestibulopathy (RV) (benign recurrent vertigo)^4,5^, delayed endolymphatic hydrops (DEH)^6,7^, post-traumatic endolymphatic hydrops or post-traumatic Meniere’s disease (MD)^8^. The superior semicircular canal dehiscence (SSCD) syndrome may also have its origin in the post-traumatic setting in a pre-disposed individual with thinning of the otic capsule over the superior semicircular canal^9^. It has been postulated that minor head trauma, even trivial, may be enough to elevate intracranial pressure enough to cause anatomic dehiscence under the circumstances. Post-traumatic perilymphatic leakage through oval and round window membranes resulting in a perilymphatic fistula (PLF) syndrome was widely reported in earlier literature^10^. Further appreciation of the pathophysiology in SSCD syndrome possibly has relegated a PLF syndrome to being anachronistic or at best an extremely rare phenomenon.

While post-traumatic BPPV is well described, the literature remains sparse documenting other disorders of peripheral vestibular dysfunction encountered in the post-head injury setting. In this paper we comment on the other peripheral vestibular disorders (exclusive of BPPV or “other” forms of positional vertigo) identified from our University Health Network (UHN), Province of Ontario, Workplace Safety and Insurance Board (WSIB) database.

Our study had specific goals of identifying:

- The clinical spectrum of post-traumatic peripheral vestibular disorders exclusive of positional vertigo.
- Whether an increased incidence of MD was found in the post-traumatic setting?
- If post-traumatic vestibular disorders were associated with the worker’s demographic profile, other related symptoms, severity of head injury, loss of consciousness (LOC), their profession, clinical examination and vestibular test results?

## Method

This study is a retrospective chart review of a de-identified data base, where every effort was made to assure privacy and confidentiality. All methods were carried out in accordance with relevant guidelines and regulations. University Health Network (UHN) institutional and/or licensing committee approved all experimental protocols. A waiver of consent was granted by UHN Research Ethics Board (Approval Number: 14-8093).

The medical records of head injured workers referred to the UHN Neurotology Clinic by the Province of Ontario WSIB between 1998 and 2018 with a diagnosis of a peripheral vestibular disorder were reviewed. All patients had a detailed neurotologic history and examination by the senior author including audiological and vestibular testing. Investigations included videonystagmography (VNG), cervical and ocular vestibular-evoked myogenic potentials (VEMPs) and video head impulse testing (vHIT). vHIT and oVEMP were not available for those studied early on in the series. Magnetic scleral search coil studies (MSSC’s) (forerunner of conventional vHIT) were available in a small minority of those studied earlier. Imaging included routine brain and temporal bone CT scans and/or brain MRI performed in advance or as determined by neurology/neurotology services for this multidisciplinary Designated Assessment Center (DAC) evaluation.

Normal limits for canal paresis in VNG (l-Portal VOG by Neuro Kinetics) were set at 15%. VEMPs were recorded using a commercially available evoked potentials system (ICS CHARTR EP 200, GN Otometrics, Taastrup, Denmark). Cervical (c) and ocular (o) VEMPs were elicited via air conduction using a 500 Hz tone. The stimulus was presented at 95 dB nHL (125 dB SPL). Absence of VEMP responses or an asymmetry ratio > 50% in c and oVEMP responses were considered abnormal. oVEMP amplitude > 17 mV was also considered abnormal. An abnormal vestibulo-ocular reflex (VOR) on vHIT (ICS Impulse, Otometrics, Taastrup, Denmark) was defined as the presence of corrective refixation saccade(s). Mean gains < 0.8 for the horizontal and < 0.65 for vertical canals was reflective for a VOR impairment.

Statistical analyses were performed using Microsoft Excel 2011 for Mac version 14.3.0. Descriptive statistics for continuous variables included mean and range and for categorical variables the frequencies were calculated. Chi-square test assessed significance of data difference. The level of significance was P < 0.05.

## Results

4291 workers were evaluated for workplace injury dizziness. 1105 (25.7%) workers were diagnosed with a peripheral vestibular disorder. 908 workers (82.17%) were diagnosed with historical or objective evidence for post-traumatic positional vertigo. Typical BPPV included those with posterior semicircular canal (PSCC) canalolithiasis. “Other” positional vertigo represented a heterogeneous category exclusive of PSSC canalolithiasis. In 244 workers (22%) the main presenting symptom was non-positional episodic vertigo (16 workers had combined peripheral and objective positional findings). The data presented focuses primarily on these 244 workers. Table 1 demonstrates the spectrum of peripheral vestibular diagnoses and their demographic features.

**Table 1:** Diagnosis of peripheral vestibular pathologies following head injury (n = 1105).

Main presentation	Diagnosis	Number of workers	% out of peripheral vestibular pathology (n = 1105)	% out of total data (n = 4291)	Mean age (years)	Sex	
						Male	Female
positional vertigo (n = 908)	Historical BPPV	714 (78.63%)	64.6	16.63	47.58 (16–82)	492 (68.9%)	222 (31.09)
	Typical BPPV	137 (15.08%)	12.3	3.19	50.81 (21–82)	104 (75.9%)	33 (24.08%)
	“Other” positional vertigo	57 (6.27%)	5.15	1.32	46.31 (20–75)	44 (77.1%)	13 (22.80%)
	Total	908	82.1	21.1	48.23 (16–82)	640 (70.4%)	268 (29.5%)
Non-positional vertigo (n = 244)	RV	78	7.05	1.81	46.3 (19–75)	45 (57.6%)	33 (42.3%)
	Uncompensated fixed vestibular loss	77	6.9	1.79	48.2 (19–75)	56 (72.7%)	21 (27.2%)
	Meniere’s disease	11	0.99	0.25	49.3 (39–70)	9 (81.8%)	2 (18.18%)
	DEH	10	0.9	0.23	45.4 (23–59)	7 (70%)	3 (30%)
	Drop attacks	9	0.81	0.20	46.7 (42–69)	5 (55.6%)	4 (44.4%)
	SSCD	3	0.27	0.06	38.3 (24–62)	2 (66.6%)	1 (33.3%)
	Undiagnosed peripheral	56	5.0	1.28	44.6 (21–70)	39 (71%)	16 (29%)

*BPPV* benign paroxysmal positional vertigo, *RV* recurrent vestibulopathy, *DEH* delayed endolymphatic hydrops, *SSCD* superior semicircular canal dehiscence.

**Figure 1:**
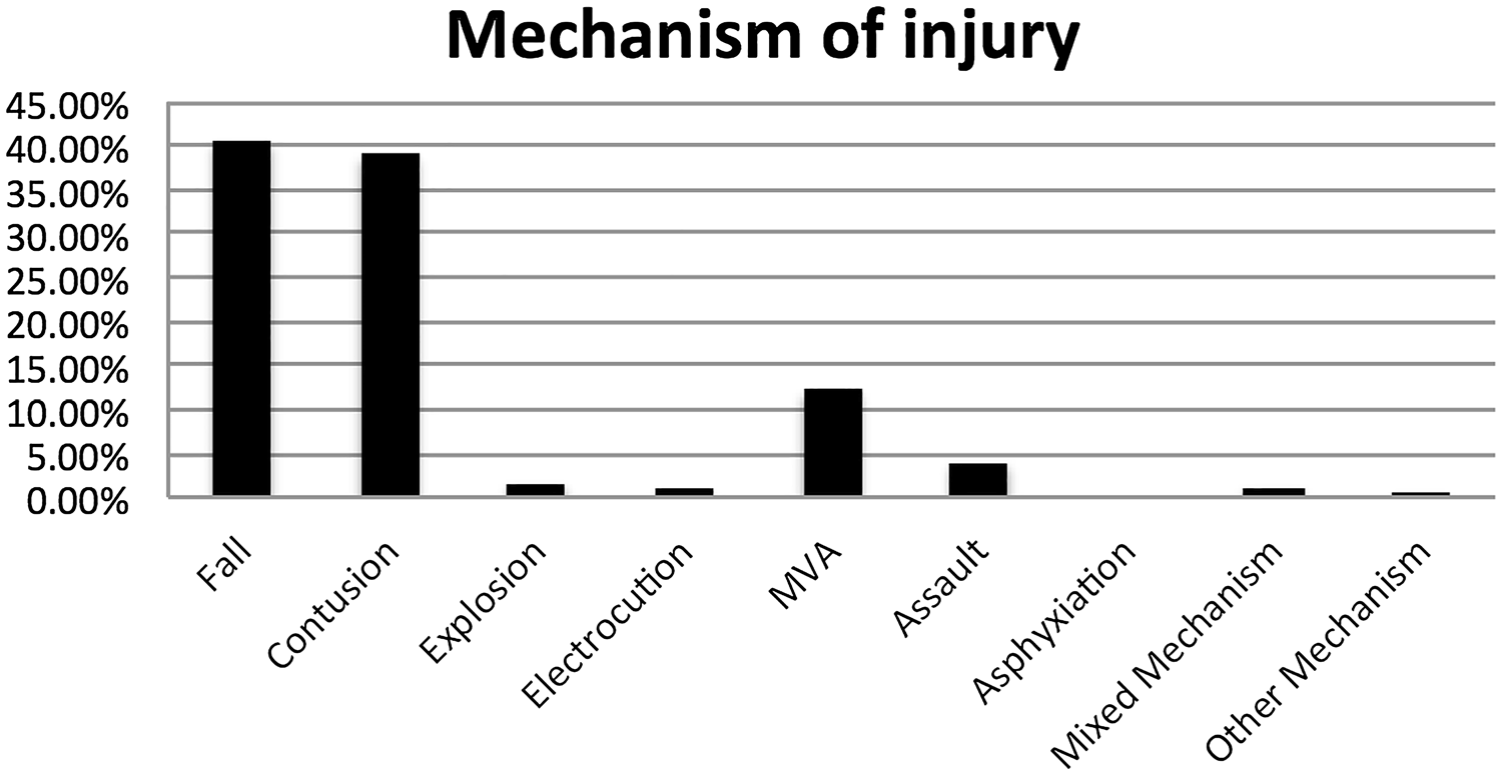
Mechanism of head injury (n = 244).

244 workers had episodic non-positional vertigo. Mean age was 46.3 years (19–79 years); male:female ratio was 164:80 (2:1). Mean time to assessment was 23.7 months (2–439 months). Common mechanisms of injury were falls (n = 99, 40.5%) and contusions (n = 95, 39%). Falls occurred from heights/slips/trips typically. The spectrum of contusions ranged from a simple bump to the head to severe head injury with permanent neurological sequelae. 30 workers (12.2%) suffered head injury from motor vehicle accidents (MVA). Figure 1 demonstrates the different mechanisms of injury.

In terms of severity the majority (78.6%) of head injured workers sustained a minor head injury or mild traumatic brain injury (mTBI) (Table 2).

**Table 2:** Severity of head injury.

Type of traumatic brain injury*	Number of patients
Minor head injury +	192 (78.60%)
Closed head injury	17 (7%)
Closed head injury + skull fracture	32 (13%)
Open/compound skull fracture	1 (0.4%)
Closed head injury + skull fracture + CSF leak	1 (0.40%)
Unknown	1 (0.40%)
Total	244 (100%)

*CSF* cerebrospinal fluid.

*According to the 2016 US Veterans Administration/Department of Defense (VA/DoD) Clinical Practice Guideline^45^ a Traumatic Brain Injury (TBI) is defined as a traumatically induced structural injury and/or physiological disruption of brain function as a result of an external force and is indicated by new onset or worsening of at least one of the following clinical signs immediately following the event: (a) Any period of loss or a decreased level of consciousness, (b) Any loss of memory for events immediately before or after the injury (post-traumatic amnesia), (c) Any alteration in mental state at the time of the injury (i.e. confusion, disorientation, slowed thinking, alteration of consciousness/mental state), (d) Neurological deficits (e.g. weakness, loss of balance, change in vision, praxis, paresis/plegia, sensory loss, aphasia) that may or may not be transient, (e) An associated intracranial lesion (typically on imaging).

^+^Numerous guidelines have been written on the inclusion criteria for diagnosis of a mild traumatic brain injury (mTBI) or minor head injury. Most concur that a mTBI (while recognizing it as a complex pathophysiological process affecting the brain) may or may not be associated with a loss of consciousness (LOC), clinically is associated with a Glasgow Coma Scale (GCS) of 13–15, resolution of post-traumatic amnesia within 24 h, a LOC for less than 30 min and normal intracranial imaging (CT/MRI)^17,46,47^.

**Figure 2:**
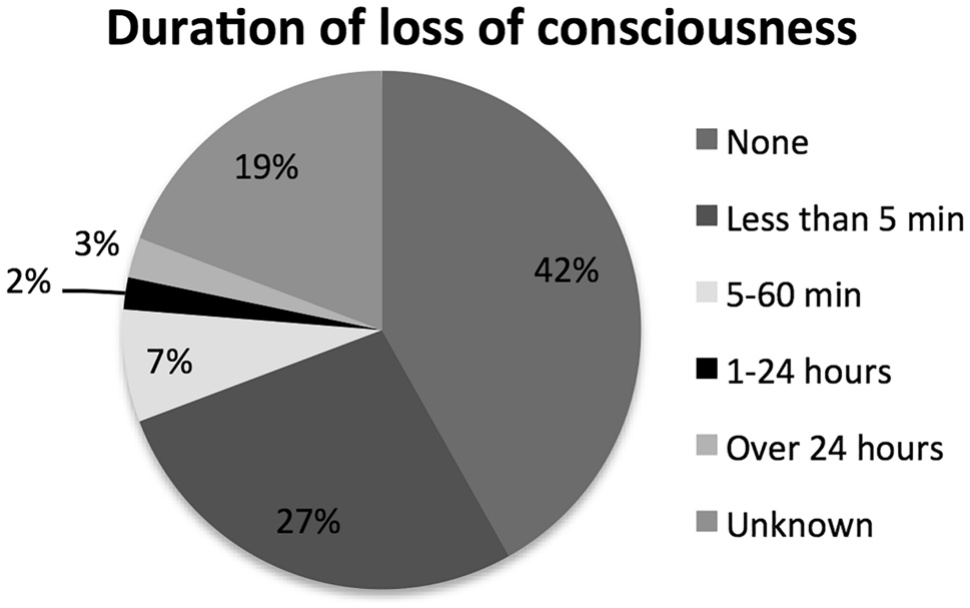
Duration of loss of consciousness following head injury.

102 workers (41.8%) with a peripheral vestibular disorder (excluding positional vertigo) sustained a mTBI without LOC. Of workers with a LOC, the majority had a loss < 5 min duration (n = 67, 41.4%). Figure 2 documents LOC in this head injury series.

The most common occupations with a peripheral vestibular disorder came from the construction industry (46 patients) and transportation services (38 patients). See Appendix, Supplementary Tables 1, 2 and Figs. 1, 2 for information on the mechanism and severity of head injury and sex predilection in patients outside of a peripheral vestibular disorder.

On subgroup analysis, there was no significant difference in mean age and mechanism of injury among different groups with peripheral vestibular disorders (Tables 1, 3). Males predominated in all subgroups. Workers with RV or drop attacks were more frequently female. In those with an uncompensated fixed peripheral vestibular loss, LOC was significantly greater than those with RV (P = 0.007) (see Table 4).

**Table 3:** Mechanism of head injury in the common types of peripheral vertigo.

Category	Injury mechanism							
	Fall	Contusion	MVA	Assault	Electrocution	Explosion	Mixed mechanism	Other mechanism
RV (n = 49)	21	21	6	1	–	–	–	–
RV-otolithic based (n = 29)	10	11	6	1	1	–	–	–
DEH (n = 10)	2	5	3	–	–	–	–	–
Drop attack (n = 9)	6	3	–	–	–	–	–	–
Definite Meniere’s (n = 6)	2	3	1	–	–	–	–	–
Probably/possible Meniere’s (n = 5)	1	2	2	–	–	–	–	–
Uncompensated fixed vestibular loss (n = 77)	26	29	9	6	1	3	2	1
SSCD (n = 3)	2	–	1	–	–	–	–	–
Undiagnosed peripheral (n = 56)	29	21	2	2	–	1	1	–
Total (n = 244)	99	95	30	10	2	4	3	1

*RV* recurrent vestibulopathy, *SSCD* superior semicircular canal dehiscence, *DEH* delayed endolymphatic hydrops. Undiagnosed peripheral disorder represented those with a history suggestive for peripheral vestibular localization but defied classification into a recognized subgroup according to symptoms/signs/duration of vertigo etc.

**Table 4:** Loss of consciousness following head trauma in the presenting types of peripheral vestibular disorders.

Diagnosis	Loss of consciousness					
	None	< 5 min	5–60 min	Unknown	1–24 h	> 24 h
RV* (n = 49)	30	10	5	4	–	–
RV-Otolithic based (n = 29)	14	9	–	5	–	1
DEH (n = 10)	4	2	2	2	–	–
Drop attack (n = 9)	5	1	2	1	–	–
Definite Meniere’s (n = 6)	3	3	–	–	–	–
Probable/possible Meniere’s (n = 5)	4	1	–	–	–	–
Uncompensated fixed vestibular loss* (n = 77)	27	23	5	20	–	2
SSCD (n = 3)	2	–	–	1	–	–
Undiagnosed peripheral (n = 56)	13	17	3	14	5	3
Total (n = 244)	102	67	17	47	5	6

*Significant difference (p < 0.05) in duration of LOC in RV vs. uncompensated fixed vestibular loss.

*RV* recurrent vestibulopathy, *SSCD* superior semicircular canal dehiscence, *DEH* delayed endolymphatic hydrops. Undiagnosed peripheral disorder represented those with a history suggestive for peripheral vestibular dysfunction but defied classification into a recognized subgroup according to symptoms/signs/duration of vertigo etc.

The severity of injury on clinical exam was not significantly different between subgroups (see Table 5).

**Table 5:** Severity of head injury following head trauma in the common types of peripheral vertigo.

Category	Severity of injury					
	Minor head injury	Closed head injury	Closed head injury + skull fracture	Open/compound skull fracture	Closed head injury + skull fracture + CSF leak	Undiagnosed peripheral
RV (n = 49)	44	3	2	–	–	–
RV-Otolithic based (n = 29)	23	1	5	–	–	–
DEH (n = 10)	7	–	3	–	–	–
Drop attack (n = 9)	8	1	–	–	–	–
Definite Meniere’s (n = 6)	6	–	–	–	–	–
Probable/Possible Meniere’s (n = 5)	5	–	–	–	–	–
Uncompensated fixed vestibular loss (n = 77)	60	7	8	1	–	1
SSCD (n = 3)	2	–	1	–	–	–
Undiagnosed peripheral (n = 56)	37	5	13	–	1	–
Total (n = 244)	192	17	32	1	1	1

*RV* recurrent vestibulopathy, *SSCD* superior semicircular canal dehiscence, *DEH* delayed endolymphatic hydrops. Undiagnosed peripheral represented those with a history suggestive for peripheral vestibular dysfunction but defied classification into a recognized subgroup according to symptoms/signs/duration of vertigo etc.

### Otological symptoms and findings

201 workers (82.37%) had associated otologic complaints; 61 (30.3%) specifically complained of hearing loss, 110 (54.7%) tinnitus and 11 (5.47%) provided a history for hyperacusis. 16 workers (7.9%) complained of aural fullness and 3 (1.49%) had otalgia.

233 workers (95.49%) had normal external auditory canals/tympanic membranes (TM’s). 8 workers (3.27%) had classic step deformities from a basal skull fracture, 2 workers (0.81%) had incidental findings of cholesteatoma and 1 worker (0.4%) had a central TM perforation.

Pure tone audiometry was normal in 90 workers (36.88%). 60 workers (24.5%) had trauma-related sensorineural hearing loss (SNHL) of varying degrees, 8 (3.27%) had a conductive component and 5 (2.04%) had a mixed (sensorineural and conductive) hearing loss. In 78 workers (31.96%) audiometric findings were typical for noise induced hearing loss and/or presbycusis unrelated to the subject accident.

### Clinical spectrum of peripheral vestibular disorders

The most common peripheral vestibular disorder identified in 4291 head injured workers was positional vertigo, either historical, typical BPPV or “other” positional type (n = 908, 21.1% of series total). PSCC canalolithiasis was identified in 137 (15.08%). “Others” had positional vertigo from different inner ear pathologies in 57 (6.27%). 714 (78.63%) workers had a characteristic history for BPPV, but could not be confirmed objectively in the Dix–Hallpike maneuver.

108 workers (2.5%) experienced recurrent attacks of episodic vertigo lasting minutes to hours. Workers were further subcategorized into RV, MD and DEH. Those with drop attacks were categorized separately. 78 (1.81%) workers without hearing loss/tinnitus or focal neurologic signs/symptoms were clinically diagnosed with RV. Features were primarily suggestive for semicircular canal involvement (attacks of spinning/rotational vertigo) in 49 workers. Episodic rocking or translational pulsion was suggestive for an otolithic type of RV in 29. Eleven workers (0.25%) fulfilled the clinical criteria for MD (6 definite and 5 probable/possible) according to the 1995 American Academy of Otolaryngology-Head and Neck Surgery (AAO-HNS) Guidelines^11^. In 10 workers (0.23%) attacks of vertigo were preceded by a unilateral post-traumatic SNHL and were diagnosed with DEH. Nine workers (0.20%) reported attacks of unprovoked falls consistent with drop attacks.

An uncompensated fixed peripheral vestibular loss was diagnosed in 77 workers (1.79%). All had persistent complaints of movement-aggravated dizziness with a significant unilateral peripheral vestibular loss on laboratory testing. While symptomatic, all had a stable peripheral vestibular loss. Temporal bone fracture was identified in 4 of this group. Trauma from contusion or a shearing type injury to the inner ear was the postulated cause for the vestibular loss.

Three (0.06%) workers had symptoms/signs for a SSCD syndrome. Laboratory findings included a conductive component to hearing, supranormal bone conduction thresholds and oVEMP amplitudes > 17 μV in our testing laboratory. All were confirmed on high resolution CT of the temporal bones to have a SSCD.

Fifty-six (1.29%) workers had peripheral vestibular symptoms and in some instances abnormalities on their clinical examination and vestibular testing suggestive for organic pathology but could not be categorized.

Of 244 workers with a peripheral vestibular disorder, 16 had temporal bone fractures. Six required neurosurgical intervention (i.e. elevation of a depressed facture/intracranial hematoma drainage). Two sustained traumatic facial nerve palsy. Clinical diagnoses in these 16 workers included fixed uncompensated vestibular loss in 4 and otolithic RV in 1. Eleven workers initially undiagnosed developed positional vertigo over their follow up.

### Vestibular function in peripheral vestibular disorders

Ninety-eight (40.1%) of 244 workers had abnormal caloric responses. Fifty-one workers (20.9%) had abnormal cVEMP results. vHIT results were available in 33 and were abnormal in 7 (21.2% of those tested) workers. oVEMP was available for 36 workers and was abnormal in 18 (50%).

Table 6 outlines the demographic and vestibular test results. As vHIT and oVEMP was not institutionally available until 2013, the majority of workers with peripheral vestibular dysfunction were not tested with either investigation.

**Table 6:** Vestibular testing results.

Category n = number of patients in each diagnostic category (total)	Caloric abnormality (n with abnormality/% of total in diagnostic category)	cVEMP abnormality	oVEMP abnormality	vHIT abnormality
Definite Meniere’(n = 6)	2 (33.3%)	1 (16.6%)	1/1*	0/1*
Probable/possible Meniere’s (n = 5)	1 (16.6%)	1 (20%)	1/1	1/1
RV (n = 49)	21 (42.85%)	6 (12.24%)	3/15	1/15
RV-otolithic based (n = 29)	6 (20.68%)	12 (41.37%)	4/7	1/7
DEH (n = 10)	5 (50%)	2 (20%)	1/1	1/1
Drop attack (n = 9)	5 (55.5%)	1 (11.1%)	0/2	0/2
Uncompensated fixed vestibular loss (n = 77)	38 (49.3%)	18/59	3/4	0/4
SSCD (n = 3)	0	1 (33.3%)	3/3	1/3
Undiagnosed peripheral (n = 56)	20 (36.3%)	8 (14.5%)	2/8	2/8

*RV* recurrent vestibulopathy, *SSCD* superior semicircular canal dehiscence syndrome, *DEH* delayed endolymphatic hydrops. Undiagnosed peripheral represented those with a history suggestive for peripheral vestibular dysfunction but defied simple classification into a recognized subgroup according to symptoms/signs/duration of vertigo etc.

*The number represents the total number of patients being tested.

On subgroup analysis, cVEMP abnormalities were more common in otolithic RV compared to semicircular canal RV [statistically significant (P = 0.03)]. Those with semicircular canal RV had a significantly higher caloric excitability difference (P = 0.046).

50% of workers with DEH had abnormalities on caloric testing at the time of their assessment. Their SNHL varied from moderate to profound.

cVEMP response thresholds were normal in all 3 workers with a SSCD.

See appendix, Supplementary Table 3 to compare vestibular testing results in patients with peripheral vestibular disorders vs. those without.

### Natural progression

Most patients were seen every 6–12 months in review if there were specific concerns (i.e. diagnosis or persistence of symptoms). The average follow-up was 20 months (range 4–99 months). Table 7 provides natural progression information within specific categories with episodic vertigo or drop attacks. Sixty-six workers (27%) were additionally diagnosed with significant anxiety or depressive symptoms requiring ongoing psychiatric/psychologic care.

**Table 7:** Natural progression.

Diagnosis	Time between injury and last follow up (months)	Active symptoms	Symptomatic improvement	Conversion to Meniere’s	Associated BPPV	Remission	Lost to follow up
Recurrent vestibulopathy (n = 49)	14.4 (6–63 months)	18 (37.5%)	15 (31.3%)	0	1 (2.1%)	9 (18.7%)	6 (12.5%)
Otolithic recurrent vestibulopathy (n = 29)	23.7 (6–289 months)	4 (13.8%)	10 (34.5%)	0	5 (17.2%)	2 (6.9%)	8 (27.6%)
Drop attacks (n = 9)	20.6 (6–52 months)	5 (55.5%)	3 (33.3%)	NA	NA	1 (11.1%)	0
Delayed endolymphatic hydrops (n = 10)	65.9 (6–439 months )	5 (50%)	3 (30%)	NA	NA	1 (10%)	1 (10%)
Meniere’s disease (n = 11) (definite and probable/possible)	27.9 (6–43 months)	5 (45.5%)	3 (27.3%)	NA	1 (9.09%)	1 (9.1%)	2 (18.2%)

## Discussion

Dizziness, headache and neurocognitive/neuropsychological dysfunction are commonly reported symptoms after head injury. This as much seems agreed.

Much has been written about mTBI following concussion and sports related injury. Large scale population studies addressing dizziness from workplace head injury does not exist nor been appropriately studied. Longitudinal outcomes for dizziness post-head injury are generally not available, excepting in certain select patient series (i.e. post-traumatic BPPV). There is significant controversy over the terminology (i.e. the use of dizziness vs. vertigo) and whether certain conditions such as post-traumatic endolymphatic hydrops, cervicogenic vertigo and traumatic PLF exist or whether their reported incidence has been inappropriately amplified through a lens of personal bias. The interplay between traumatic vestibular dysfunction and associated non-organic (i.e. mood change, functional amplification, catastrophization etc.) features also requires careful consideration. Both may converge and adversely influence each another including other workplace considerations (i.e. return to a toxic work environment).

A deliberate decision was made to exclude those with symptoms likely reflective for cognitive vestibular dysfunction. Euphemistic terms such as medically unexplained dizziness, psychogenic dizziness, non-organic dizziness and chronic non-specific dizziness have all been applied. Predisposing circumstances and specific symptoms absent of fluctuant vestibular activity have received diagnoses such as mal d’embarquement, chronic phobic dizziness, persistent perceptual postural dizziness (PPPD) etc. While a traumatic vestibular injury may have been the epiphenomenon, the persistent downstream complaints strongly indicate another generator. Vestibular rehabilitation therapy (VRT) seems of limited value. Treatment with mood stabilizing medications (i.e. the SSRI or SNRI classes of antidepressant therapy) and interventions such as cognitive behavioral therapy seem likely to be recommended.

All 4291 head injured workers had had complaints of dizziness as their point of entry (see Appendix, Supplementary Fig. 3 for diagnostic groups in the whole database). Of those approximately 25.7% (1105 head injured workers) were identified with evidence suggestive for a peripheral vestibular disorder. The majority (82.17%) were identified with positional vertigo based on history, subjective complaints during testing or objective findings on examination. Not all with BPPV had findings for PSCC canalolithiasis. The “other” positional vertigo category was designated for positional vertigo involving other semicircular canals, if cupulolithiasis was present, if nystagmus patterns atypical for canalolithiasis/cupulolithiasis were identified (i.e. downbeat nystagmus) and those rarely arising from a central vestibular localization.

The remaining 22% (244 head injured workers) diagnosed with a recurrent peripheral vestibular disorder was the subject of this paper. Diagnostic entities included recurrent attacks of episodic vertigo identified as RV, post-traumatic MD, DEH, drop attacks (so-called “crisis of Tumarkin”), trauma associated SSCD and undiagnosed (unknown) peripheral vestibular disorders.

### Recurrent vestibulopathy (78/244)

RV was diagnosed in 78 of 244 (35%) head injured workers. By definition RV denotes individuals with recurrent bouts of spontaneous episodic vertigo lasting minutes to hours (similar to MD) in the absence of auditory or focal neurologic dysfunction^4,5^. Episodes of rotational type vertigo were primarily attributed to semicircular canal dysfunction. Those with complaints of episodic rocking, imbalance or translational pulsion arose from an otolithic source^1,4^.

In Rutka and Barber’s^4^ longitudinal RV study of 86 non-head injured patients over 8.5 years, 62% had complete resolution of symptoms, 9.5% continued to have active episodes, 13.5% evolved into MD and 8% developed BPPV. A further 7% remained undiagnosed but had continued symptoms for a peripheral vestibular localization. In the current study group, no head injured worker with RV evolved to MD. Most were female. Mean presentation age did not differ significantly between RV and Meniere’s overall.

Five patients (17.2%) with otolithic RV developed BPPV compared to only 1 patient (2.1%) with a semicircular origin. Detachment of otoconia from the utricular neuroepithelium into the semicircular canal circulation that float freely or attach to the cupula seemed likely to account for positional vertigo from canalolithiasis or cupulolithiasis^12^. Workers with otolithic RV had a greater likelihood of developing concomitant BPPV compared to those with a semicircular canal RV origin. Long-term follow up is needed to determine whether both variant phenotypes might evolve to other identifiable inner ear disorders.

In the absence definitive pathophysiology RV has been ascribed in certain circles to post-traumatic headache (PTH) or as an equivalent of vestibular migraine (diagnostic criteria specific for vestibular migraine were formally established by Barany society in 2012^13^ and added into the International Classification of Headache Disorders in 2013^14,15^). In our opinion both unfortunately fail to account for differences and further complexities in those with head injury. Headache along with neuropsychological/neurocognitive change commonly intersected in 3884 (90.5%) and 3189 (74.3%) of the 4291 head injured workers respectively. In those with RV, 93.2% had significant complaints of headaches (including migraine) in this series. Headache was also the most commonly associated symptom in those with a peripheral vestibular disorder. All 11 (100%) workers with MD conversely reported headache more frequently than those with RV.

When deep phenotyping evaluated PTH in one series, 87 of 100 individuals with mTBI had concomitant complaints of dizziness/vertigo^16^. Chronic migraine-like headache occurred in 61 and a combination of episodic migraine and tension-type headache in 29. Difficulty however arose in determining whether headache and dizziness together were causally related or represented an intersection between two common post-head injury symptoms. Therapeutic migraine-specific preventative mediations seemed to lack efficacy overall^16^. Trauma as the sole cause for migraine development also seemed poorly correlated to severity of injury and other factors such as an individual’s pre-morbid emotional status, past history of motion sickness and positive family history^17^.

By definition, in vestibular migraine, the migraine headache should temporally coincide largely with a vertiginous attack. Inclusion of an “amigrainous” vertigo category (episodic vertigo without headache) seems to further blur what we categorized as RV. In our opinion the commonality of PTH (including migraine) meant we could not reliably differentiate migraine especially as a cause for recurrent attacks of peripheral vestibular dysfunction.

### Meniere’s (definite and probable/possible) (11/244)

Post-traumatic MD was rare in our series. In earlier literature, Paparella and Mancini hypothesized trauma could cause mechanical or biochemical changes within the inner ear leading to the dysfunction endolymph producing cells and/or affect mechanisms of endolymph absorption. The latter mechanism resulted in Meniere’s syndrome from post-traumatic hydrops. Nearly 2/3rd of their cohort with physical trauma developed Meniere’s syndrome one or more years later. One third of their patients however presented within 1 month of injury^8^. In another series with 120 MD patients, less than 3% were found to have the condition arise from trauma^18^. MD additionally has been reportedly associated with temporal bone fracture^19,20^.

A diagnosis for formal MD was based on criteria put forth by the 1995 AAO-HNS CHE Guidelines^11^.

A total of 11 patients (0.26%) were diagnosed (6 definite and 5 probable/possible) with MD. Since 2017, no new patients have surfaced with this diagnosis^1^. The incidence of MD from the UHN WSIB database at 0.25% seems comparable to a quoted incidence of 0.2% in the general population^21,22^. Accordingly one cannot conclusively state that Meniere’s disease/syndrome arises in a greater proportion following head injury compared to its occurrence in the general population. This also seems in accord with the reported incidence of MD by Segal et al. in their study of Israeli Defence Force (IDF) personal post acute acoustic trauma^23^. See Appendix, Supplementary Table 4.

The association between BPPV and Meniere’s however is well recognized and some studies have shown that about 1/3 with MD can develop concomitant BPPV^24^. The high prevalence of BPPV in MD is generally explained by pathophysiological injury to the otolithic organs with release of displaced otoconia /particulate debris into the PSCC circulation.

Most with MD received conservative medical treatment typically with diuretics or betahistine (Serc)^®^ (BGP pharma ULC, Etobicoke, Ontario). To date, only one patient with established Meniere’s disease has developed concomitant BPPV. Of 9 patients who visit us regularly 5 remain symptomatic. Differences in the natural history for post-traumatic MD versus MD from idiopathic endolymphatic hydrops would require further long-term prospective cohort studies with larger numbers in the former.

### Drop attacks (9/244)

Drop attacks occurred in 9/244 (3.7%) workers with peripheral vestibular dysfunction. Tumarkin falls (“crisis of Tumarkin”) or sudden drop attacks are typically seen in a subset of patients with MD. Falls are sudden and occur without warning, LOC or concomitant neurologic symptoms. They are likened to a feeling of being pushed/pulled or arise from an unanticipated loss of postural tone linked to vestibular causes^25^. They have also been associated with vertiginous disorders such as a SCDS^26^ and other peripheral vestibulopathies^27^.

Non-Meniere’s drop attacks with associated recurrent spontaneous vertigo spells have been described in a previous case series of 6 patients^28^. No patient had had a history of head trauma. There was an association with migraine mentioned in 5 of 6 cases interestingly. The authors however concluded that an etiologic link between the two could not be established. In our study, drop attacks were observed in 9 patients who did not meet the criteria for MD. None specifically complained of major vertiginous attacks.

The natural history of vestibular drop attacks has not reported extensively. Treatment remains a matter of pointed discussion; most cases having a benign course and a high rate of spontaneous remission^28,29^. Intratympanic (IT) dexamethasone injections have been promoted with a success rate of 70% quoted^30^. In severe cases where falls might be associated with further head injury or fractures more aggressive management maybe necessary. Under these circumstances IT gentamicin reportedly achieved success rates from 60 to 100% controlling drop attacks in MD^31^. More invasive intervention (i.e. total osseous labyrinthectomy) has been studied in the elderly and reported to have provided good symptom control^32^.

All patients with drop attacks were managed conservatively (mean 20.6 months). Five workers continued to have drop attacks with the same severity, 3 had some improvement with respect to frequency of their drops and 1 patient has remained free of attacks for one-year’s duration. As less seems known about the progression of these attacks and whether certain individuals might warrant more aggressive treatment, the debate for best management strategy remains open.

### Delayed endolymphatic hydrops (10/244)

Schuknecht^6^ generally receives credit for clinically differentiating and for the histopathology study of DEH in this related condition to MD. On clinical grounds DEH is diagnosed in patients with a significant SNHL (secondary to trauma, surgery, infection etc.) who later develop attacks of episodic vertigo similar in duration to MD. Attacks of vertigo can arise from the side with the hearing loss (ipsilateral DEH) or rarely, the contralateral side (contralateral DEH)^6^.

Head trauma is a well-established predisposing factor for DEH. The latent timeframe for developing vertiginous attacks can be as early as one month from inner ear injury^7,8^ to many years later. The proposed hypothesis assumes a labyrinthine insult strong enough to cause a significant hearing loss but simultaneously preserves some vestibular function; over a period of time atrophy or fibrous obliteration of endolymphatic system could lead to episodes of vertigo on the basis of hydrops^33^.

Treatment options include conservative management, IT therapies (both steroid and gentamicin) and surgical labyrinthectomy^6,34^. Workers with DEH had a mean follow up period of 65.9 months. All were managed conservatively. About 30% had symptomatic improvement, 10% had no symptoms over their last 6 months of follow up while 50% continued to be symptomatic. Resolution of the vertiginous attacks in this series differs from those reported by Kamei and Matsuzaki, who observed that about 65% of their patients with DEH had resolution of symptoms managed conservatively within 5 years of symptom onset^7^. 

### SSCD (3/244)

A SSCD syndrome has been identified in some following head injury. Carey et al. suggested that approximately 2% of individuals in the general population likely have a dehiscence or very thin bone overlying the superior canal^9^. An event such as a closed head injury causing transient elevations in intracranial pressure may be all that is required to fracture the thin bone overlying the superior canal or destabilize its dural covering over a pre-existing dehiscence, leading to a symptomatic dehiscence syndrome^35^.

Our series had 3 workers with recognized symptoms and audiovestibular characteristics for a SCCD. All had significantly elevated oVEMP amplitudes (> 17 μV). Temporal CT imaging confirmed the dehiscence in all 3. There was no history/symptoms referable to a SSCD syndrome prior to head injury. None have warranted surgical intervention at the time of writing.

### Uncompensated fixed vestibular loss (77/244)

A significant peripheral vestibular loss was diagnosed and confirmed on laboratory testing in 77 workers. Mechanisms for injury included temporal bone fracture (4 workers) or from suspected shearing type injuries within the inner ear. Acute vertigo from labyrinthine injury usually resolved over a period of weeks or months^3^. Not all patients however following a vestibular loss seemed to compensate equally. Failure to compensate from a fixed uncompensated vestibular loss remains another cause for movement aggravated dizziness post head injury. While vestibular suppressants may dramatically improve symptoms in the acute phase of vestibular loss, continued use can delay compensation and subsequent recovery^36^. VRT remains the mainstay treatment by enhancing vestibular plasticity. Failure to compensate raises concerns regarding whether existing pathology involves the contralateral ear (i.e. in a coup and contrecoup type injury). Functional amplification of symptoms from anxiety or the development of an underlying mood change may also adversely affect the compensation process^37^.

### Undiagnosed peripheral (55/244)

Approximately 23% of head injured workers initially had signs/symptoms suggestive for peripheral vestibular dysfunction but could not be formally categorized into a well-recognized disorder or syndrome. Whether there could be evolution to a more recognizable vestibular disorder remains to be determined. Some 31 head injured workers were later demonstrated to have findings attributable to positional vertigo.

The major strength of this study is found in the large number of workers who were comprehensively examined and investigated by the senior author from 1988–2018 in a multidisciplinary DAC approach. The collection of “big data” has allowed further insight into the presenting symptoms and spectrum of peripheral vestibular disorders seen post-head injury exclusive of post traumatic positional vertigo. Continued review especially in those with “*Other” Positional Vertigo* or a*n Undiagnosed* peripheral vestibular disorder will help determine the natural progression and/or evolution towards a more recognizable forms of vestibular dysfunction over time. Whether our findings can be translated to TBI’s involving other professions such as the military (conflict vs. non-conflict generated) or TBI’s associated with other activities including those sports related (professional vs. recreational generated) seem grounds for further study.

Weaknesses in this study reside in the usual criticisms applied to any retrospective study. All head injured workers were also reviewed within a single institutional setting by the senior author. Consideration for diagnostic bias might limit the study’s generalizability. New vestibular testing beyond conventional ENG/rotational chair also became available during the study meaning that VEMP and vHIT was not available for head injured workers early on. Longterm outcome data was also not available for most and whether those with a peripheral vestibular disorder ultimately had resolution or remained symptomatic cannot be answered. The burden post-traumatic dizziness places on both the worker and society at large cannot be answered. The exclusion or failure to identify/include what we considered clinical entities not without controversy such as post-traumatic PLF, cervicogenic vertigo, post-traumatic vestibular migraine (including amigrainous vertigo) etc. may also be a point of concern to some. Diagnostic entities that largely have a persistent non-organic basis increasingly being referred to as syndromes of cognitive vestibular dysfunction (i.e. chronic subjective dizziness^38^, PPPD^39,40^, chronic phobic postural dizziness^41^, medically unexplained dizziness^42^, dizziness with catastrophization^37^, mal d’embarquement^43,44^ etc.) were also excluded despite acknowledgement an acute vestibular event might be a precipitating cause.

## Conclusion

Based on a large database of 4291 head injured workers with dizziness, 1105 (25.7%) were identified to have a suspected peripheral vestibular localization for their symptoms. Positional vertigo was suspected or confirmed in 908 head injured workers or 82.1%. 244 or 22% of head injured workers were diagnosed with non-positional vertigo arising from a peripheral vestibular disorder and was the subject of this paper. The clinical spectrum pertaining to RV (semicircular vs. otolithic based), MD and the related conditions of DEH and drop attacks, post- traumatic SSCD, those with an uncompensated peripheral vestibular loss and an undiagnosed peripheral vestibular disorder has been discussed.

Patients with otolithic RV had greater chance of developing BPPV than those with semicircular canal RV. The incidence of Meniere’s disease in the post-traumatic setting did not appear greater than found in the general population. Patients with post-traumatic RV and drop attacks were more likely to be female. Patients with an uncompensated fixed vestibular loss had a higher incidence of LOC compared to those with RV. cVEMP abnormalities were significantly more common in otolithic RV compared to semicircular canal RV. Conversely those with semicircular canal RV had a significantly higher incidence of a caloric test abnormality. We could not find a strong association between the worker’s age, other associated symptoms, severity of injury and their profession for the development of a specific post-traumatic peripheral vestibular disorder.

## Supplementary Information

Supplementary Legends.

Supplementary Figure 1.

Supplementary Figure 2.

Supplementary Figure 3.

Supplementary Table 1.

Supplementary Table 2.

Supplementary Table 3.

Supplementary Table 4.
