# Supplementary material for: Post-traumatic peripheral vestibular disorders (excluding positional vertigo) in workers following head injury
Source: Sci Rep. 2021 Dec 6;11:23436. doi: 10.1038/s41598-021-02987-5 (PMC8648866; doi:10.1038/s41598-021-02987-5)
Supplement: Supplementary file 1 — Supplementary Legends. [file 41598_2021_2987_MOESM1_ESM.docx]

**Supplementary Figure 1: Mechanism of head injury in non-peripheral vestibular diagnoses (n=3186)**

**Supplementary Table 1: Severity of head injury in non-peripheral vestibular diagnoses (n=3186)**

**Supplementary Figure 2: Level of consciousness in head injury non-peripheral diagnoses (n=3186)**

**Supplementary Table 2**: **Sex differences in males vs. females following head injury**

**Supplementary Figure 3: Diagnostic Groups in Data Base of Head Injured Workers (N=4291)**

**Supplementary Table 3: Vestibular Test Abnormalities in Non-Peripheral vs Peripheral Vestibular Disorders (n=4291)**

**Supplementary Table 4: Epidemiology of Meniere’s disease**

(With acknowledgement, [Timothy C. Hain, MD](https://dizzy-doc.com/cv/hain.html) Page last modified: March 2, 2021)
