# Supplementary figures and images for: Post-traumatic peripheral vestibular disorders (excluding positional vertigo) in workers following head injury
Source: Sci Rep. 2021 Dec 6;11:23436. doi: 10.1038/s41598-021-02987-5 (PMC8648866; doi:10.1038/s41598-021-02987-5)

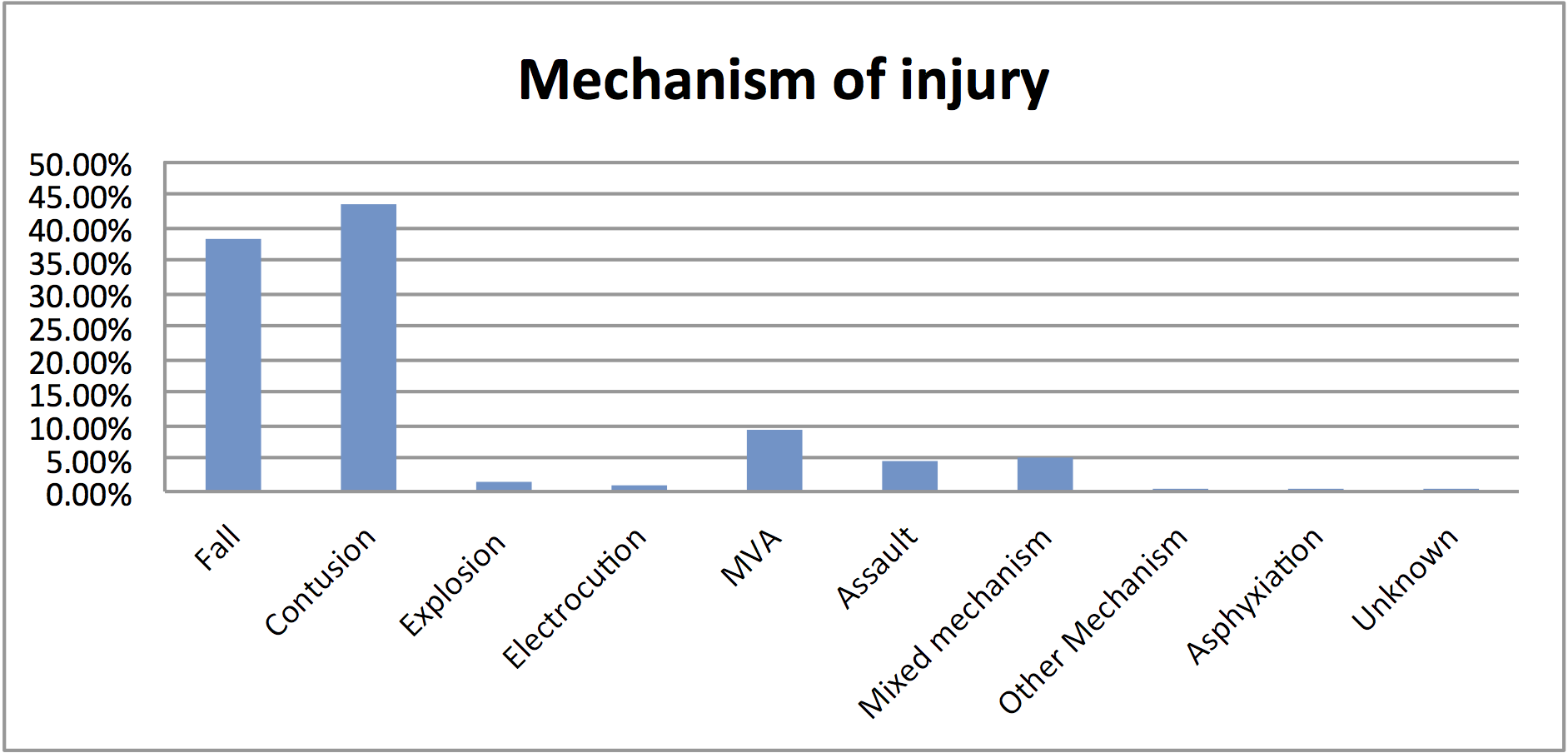

Supplement: Supplementary file 2 — Supplementary Figure 1. [file 41598_2021_2987_MOESM2_ESM.tiff]

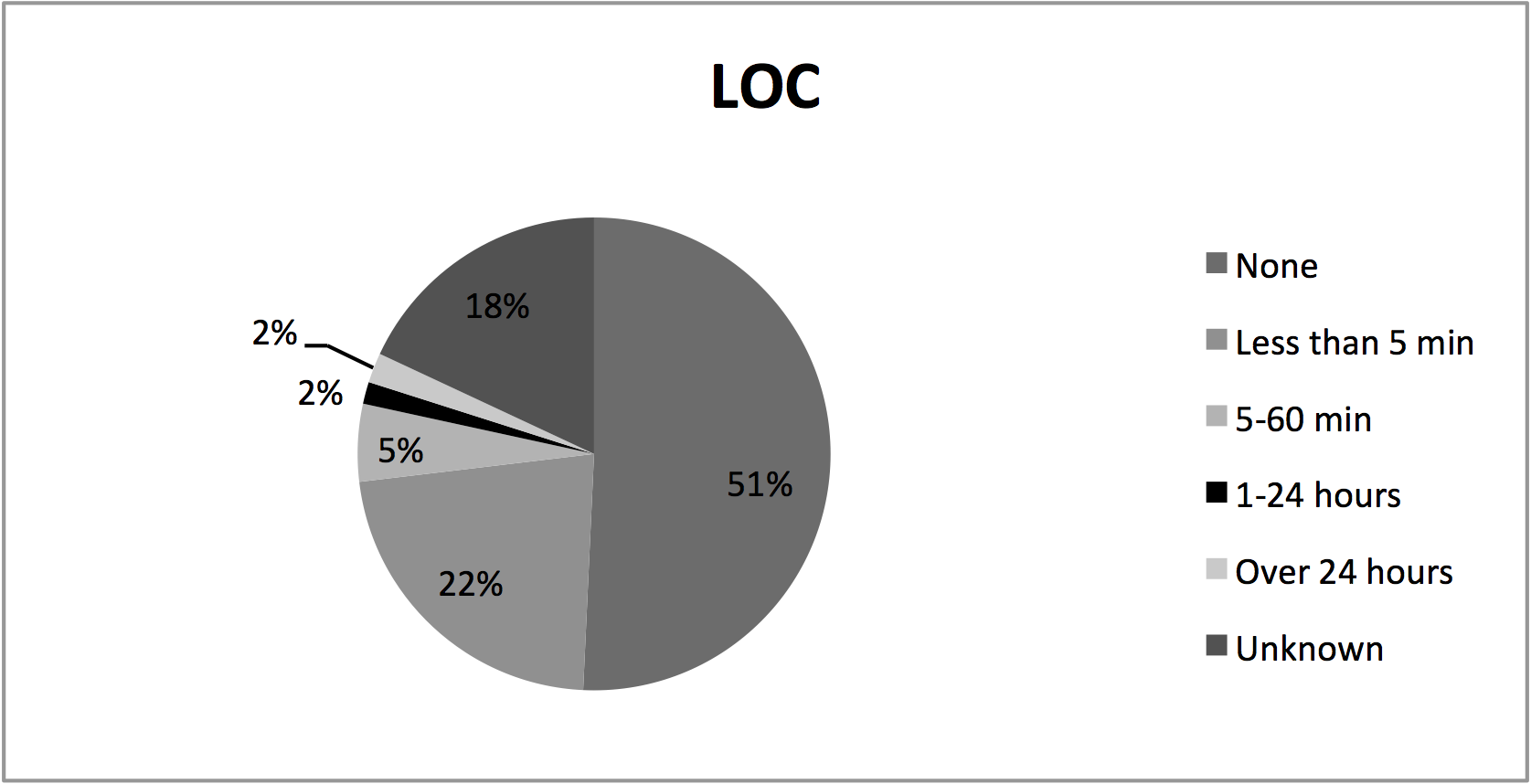

Supplement: Supplementary file 3 — Supplementary Figure 2. [file 41598_2021_2987_MOESM3_ESM.tiff]

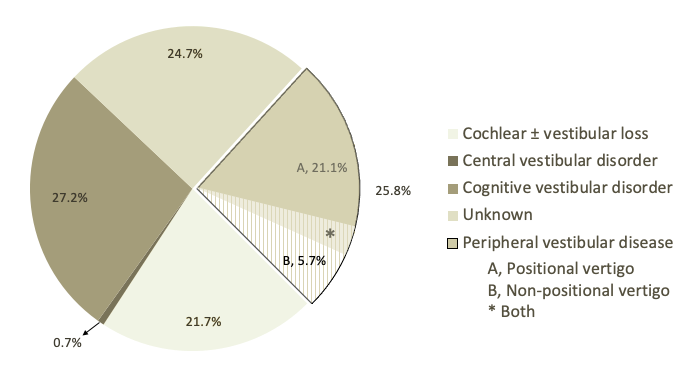

Supplement: Supplementary file 4 — Supplementary Figure 3. [file 41598_2021_2987_MOESM4_ESM.tiff]
