# Supplementary material for: Post-traumatic peripheral vestibular disorders (excluding positional vertigo) in workers following head injury
Source: Sci Rep. 2021 Dec 6;11:23436. doi: 10.1038/s41598-021-02987-5 (PMC8648866; doi:10.1038/s41598-021-02987-5)
Supplement: Supplementary file 5 — Supplementary Table 1. [file 41598_2021_2987_MOESM5_ESM.docx]

| **Type of traumatic brain injury** | **Number of patients** |
| --- | --- |
| Minor head injury | 2691 (84.46%) |
| Closed head injury | 168 (5.27%) |
| Closed head injury + skull fracture | 256 (8.03%) |
| Open/compound skull fracture | 17(0.53%) |
| Closed head injury + CSF leak | 1 (0.03%) |
| Closed head injury + skull fracture + CSF leak | 16 (0.50%) |
| Open skull fracture + CSF leak | 5 (0.15%) |
| Unknown | 32 (1%) |
| Total | 3186 (100%) |

CSF: cerebrospinal fluid
