# Supplementary material for: Post-traumatic peripheral vestibular disorders (excluding positional vertigo) in workers following head injury
Source: Sci Rep. 2021 Dec 6;11:23436. doi: 10.1038/s41598-021-02987-5 (PMC8648866; doi:10.1038/s41598-021-02987-5)
Supplement: Supplementary file 6 — Supplementary Table 2. [file 41598_2021_2987_MOESM6_ESM.docx]

|  | Male | Female | Total |
| --- | --- | --- | --- |
| Total traumatic brain injury | 2860 (66.6%) | 1431 (33.3%) | 4291 |
| mTBI | 2131 (61.4%) | 1341 (38.6%) | 3472 |
| Total peripheral vestibular dysfunction | 164 (67.3%) | 80 (32.7%) | 244 |
| Total peripheral vestibular dysfunction with mTBI | 120 (62.5%) | 72 (37.5%) | 192 |

mTBI: Mild traumatic brain injury
