# Supplementary material for: Post-traumatic peripheral vestibular disorders (excluding positional vertigo) in workers following head injury
Source: Sci Rep. 2021 Dec 6;11:23436. doi: 10.1038/s41598-021-02987-5 (PMC8648866; doi:10.1038/s41598-021-02987-5)
Supplement: Supplementary file 7 — Supplementary Table 3. [file 41598_2021_2987_MOESM7_ESM.docx]

| Vestibular testing | Caloric abnormality | cVEMP abnormality | oVEMP abnormality | vHIT abnormality |
| --- | --- | --- | --- | --- |
| Non peripheral vestibular disorders (n=3186) | 632 (19.8%)* | 603(25.9%) | 231 (31.3%) | 154 (24.6%) |
| Peripheral vestibular disorders (including positional vertigo)  (n=1152) | 303(28%)* | 215 (28.6%) | 70 (38.25) | 45 (27.1%) |

Vestibular test abnormality in non-peripheral vestibular disorders

*Chi-square test, significant
