# Supplementary material for: Post-traumatic peripheral vestibular disorders (excluding positional vertigo) in workers following head injury
Source: Sci Rep. 2021 Dec 6;11:23436. doi: 10.1038/s41598-021-02987-5 (PMC8648866; doi:10.1038/s41598-021-02987-5)
Supplement: Supplementary file 8 — Supplementary Table 4. [file 41598_2021_2987_MOESM8_ESM.docx]

| Country | Prevalence/100,000 | Incidence | Mystery number | Reference | Criteria/Comments |
| --- | --- | --- | --- | --- | --- |
| USA | 218 | 15.3 |  | Wladislavosky-Waserman et al, 1984 | Older, loose criteria |
| USA | 190 |  |  | Harris and Alexander, 2010 | Bae on 60 million patients |
| England |  |  | 180 | Cawthorne and Hewlett (1954) | Not sure if incidence or prevalence |
| England |  |  | 100 | Harrison and Naftalin, (1968) | “Clinical estimate” |
| England | 56 |  |  | Goodman (1957) |  |
| Ireland |  | 10-20 |  | Wilmot (1983) | Old, loose criteria |
| Japan | 21-36 |  |  | Shojaku and Watanabe (997), Shojaku et al, 2009 | Japanese criteria not US criteria |
| Sweden |  |  | 45 | Stahle (1973) | Not sure if incidence or prevalence |
| Finland | 43 | 4.3 |  | Kotimaki et al, (1999) |  |
| Finland | 513 |  |  | Havia et al (2005) | Helsinki |
| Italy | 205 | 8 |  | Celestino and Ralli (1991) |  |
